# Lung Fluid Volume during Cardiopulmonary Exercise Testing

**DOI:** 10.3390/medicina58050685

**Published:** 2022-05-22

**Authors:** Teruhiko Imamura, Masakazu Hori, Nikhil Narang, Koichiro Kinugawa

**Affiliations:** 1 Second Department of Internal Medicine, University of Toyama, Toyama 930-0194, Japan; masahori6059@yahoo.co.jp (M.H.); kinugawa-tky@umin.ac.jp (K.K.); 2 Advocate Christ Medical Center, Oak Lawn, IL 60453, USA; nikhil.narang@gmail.com

**Keywords:** congestion, heart failure, hemodynamics, ReDS

## Abstract

*Background and Objectives:* Cardiopulmonary exercise testing can be used to quantify exercise capacity in patients with heart failure with reduced ejection fraction (HfrEF). Lung fluid levels as measured non-invasively by remote dielectric sensing (ReDS^TM^), often correlate with intracardiac filling pressures. The change in lung fluid levels in patients with HfrEF during cardiopulmonary exercise testing is unknown. *Materials and Methods:* Patients with chronic HfrEF who underwent cardiopulmonary exercise testing between October 2021 and March 2022 were prospectively included in this proof-of-concept study, with ReDS values measured before and after testing. *Results:* Thirteen patients (median age 41 (37, 52) years, 69% men, plasma B-type natriuretic peptide 141 (57, 368) pg/mL) were included. Median peak oxygen consumption was 11.4 (10.7, 14.0) mL/kg/min. During the test, ReDS values increased from 25% to 32% only in one patient on inotropic support, whereas ReDS values remained unchanged in the other 12 patients. The former patient remained hospitalized, whereas the other patients were dischargeable without any new incidence of clinical events during the observational period (median duration 69 (33, 112] days). *Conclusions:* The ReDS system may be a feasible complementary tool to noninvasively assess the changes in lung fluid levels during cardiopulmonary exercise testing. The clinical implications of ReDS values during exercise needs further investigation.

## 1. Introduction

Cardiopulmonary exercise testing (CPET) is a well-validated risk prognostication tool in patients with chronic heart failure with reduced ejection fraction (HfrEF) for the assessment of exercise capacity [1]. Typically, a peak oxygen consumption (VO_2_) less than 12 mL/kg/min (on beta-blockers) or 14 mL/kg/min (off beta-blockers) at maximum exercise is strongly associated with worse clinical outcomes and progression to advanced heart failure [2]. One of the mechanisms of dyspnea in chronic HfrEF patients is the rise in left atrial pressure and subsequent worsening pulmonary congestion during exercise [3]. This can be quantified accurately with the use of simultaneous right heart catheterization [4], though routine implementation of invasive cardiopulmonary exercise stress testing is both challenging and resource-consuming.

**Figure 1:**
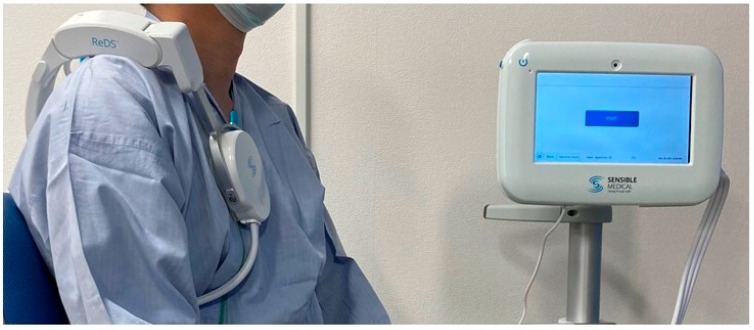
An ReDS system consists of a monitor and a sensor unit [6].

The remote dielectric sensing (ReDS^TM^, Sensible Medical Innovations Ltd., Netanya, Israel) system is a novel electromagnetic-based modality to quantify lung fluid volume noninvasively. Prior data has suggested that lung fluid volume levels measured with ReDS (Figure 1) correlate with pulmonary capillary wedge pressure in patients with HfrEF [5]. The ReDS system additionally has been shown to quantify lung fluid volume levels, with a resolution comparable to chest imaging with computerized tomography [6].

The ReDS system may be a promising tool to non-invasively estimate the change in lung fluid volumes during CPET. This additional data may offer further risk stratification, in addition to consideration of beneficial therapeutic adjustments. In this proof-of-concept study, we aimed to measure the change in ReDS values during CPET in patients with chronic HfrEF.

## 2. Methods

### 2.1. Participant Selection

Patients with chronic HfrEF who underwent CPET at our institution at clinically table condition between October 2021 and March 2022 were prospectively studied. All included patients received ReDS measurements before and after CPET, as detailed below.

Patients who were not appropriate to engage in exercise due to active diseases, including uncontrolled hypertension, severe valvular diseases, pulmonary embolism, aortic dissection, severe systemic infection, and unstable ischemic heart diseases, were not included. Those who could not engage in exercise due to sarcopenia were excluded. Patients with active intra-thoracic diseases, including pulmonary pneumonia and lung cancer, were also excluded because they might affect ReDS values.

This study was approved by the institutional review board, and all participants signed informed consent prior to study inclusion.

### 2.2. Cardiopulmonary Exercise Test

A symptom-limited CPET was performed using a bicycle ergometer with a ventilator and expired gas analyzer (Minato Medical Science, Osaka, Japan), according to the guidelines [7]. All patients initiated the test at 20 W for a 4-minute warm-up period following the initial 3-minute rest period, and underwent a 10-Watt-per-minute ramp incremental protocol.

Continuous data of VO_2_, production of carbon dioxide, and minute ventilation were measured during the tests on a breath-by-breath basis. Peak VO_2_ was defined as the highest mean VO_2_ over 20 s during the exercise.

Considering the Borg scale, which indicated patients’ objective fatigue in a range of 6–20, a score above 17 was targeted to terminate the tests. The respiratory exchange ratio was targeted at above 1.10.

### 2.3. ReDS System

ReDS values were measured just before and after CPET in all patients. ReDS employs low-power electromagnetic signals emitted between two sensors embedded in wearable devices (Figure 1). The analyzed signals reflect the dielectric properties of the lung portion. Their dielectric coefficients are represented by a frequency-dependent number describing its interaction with electromagnetic energy. As a result, ReDS estimates the lung fluid volume as a percentage with the manufacture-proposed normal range between 20% and 35%.

### 2.4. Statistical Procedures

All continuous data are presented as medians with interquartile range (IQR). Categorical data are presented as numbers and percentages. The primary aim of the study is to determine the change in ReDS values prior to and after CPET. Changes in ReDS values during exercise tests were analyzed using Wilcoxon signed-rank test. All calculations were performed with SPSS Statistics 23.0 software (IBM Corp, Armonk, NY, USA), and two-sided *p* values less than 0.05 were considered significant.

## 3. Results

### 3.1. Baseline Characteristics

Thirteen patients in total were included, with all of them completing CPET without complications. Median age was 41 (37, 52) years, and 69% were men (Table 1). Half of them had dilated cardiomyopathy. More than half of the patients had a history of heart failure admissions. All patients had reduced or mildly reduced left ventricular ejection fraction. All patients were on appropriate guideline-directed medical therapy as tolerated, with two patients requiring continuous inotropes support.

Median (IQR) peak VO_2_ was 11.4 (10.7, 14.0) mL/kg/min, and the minute ventilation/carbon dioxide production slope was 33.1 (30.9, 36.7). The procedures were performed during their hospitalization for six patients, and in outpatient clinics for the remaining seven patients.

### 3.2. Change in ReDS Values during Cardiopulmonary Exercise Tests

**Figure 2:**
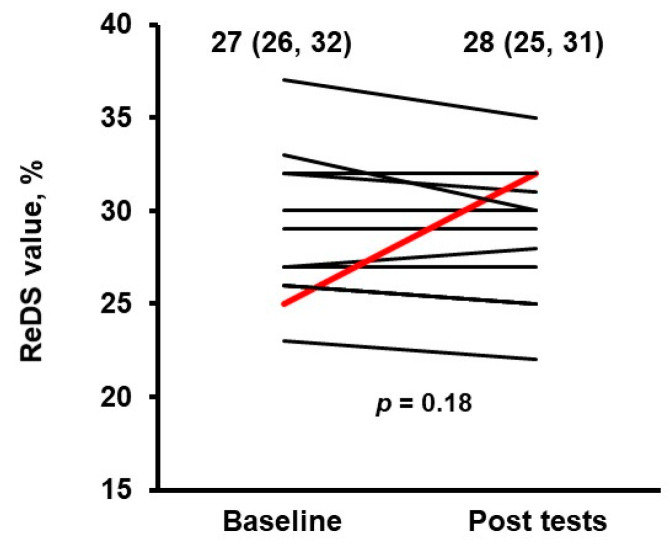
Change in ReDS values before and after cardiopulmonary exercise tests. *p* value was calculated by Wilcoxon signed-rank test.

All patients completed ReDS measurements just before and after cardiopulmonary exercise tests without any troubles and measurement errors. Overall, ReDS values did not significantly change pre- and post-CPET in nearly all patients (27% [26%, 32%] to 28% [25%, 31%]; *p* = 0.18; Figure 2). However, ReDS values increased considerably (from 25% to 32%) in one patient who was on inotropic support (Table 1). The patient was a 45 year-old man with a left ventricular ejection fraction of 33%, and left ventricular end-diastolic diameter of 75 mm. His peak VO_2_ of 16.1 mL/kg/min was higher compared to the majority of the study cohort.

### 3.3. Post-Procedure Course

The patient with incremental ReDS values during the test remained hospitalized for 34 days as of the end of this study, undergoing evaluation for heart transplantation.

Median follow-up period of the other 12 patients was 69 (33, 112) days; Of these, five patients were hospitalized at the time of testing and were eventually discharged.

## 4. Discussion

In this preliminary, proof-of-concept, prospective study, we assessed the feasibility of ReDS measurements before and after CPET in patients with chronic HFrEF, in order to quantify the change in lung fluid levels during exercise. We observed no significant change in ReDS values in the majority of patients, except for a single patient dependent on inotropic support, whose ReDS value increased from 25% to 32%.

CPET is an established diagnostic modality to objectively quantify patients’ exercise capacity in the setting of chronic heart failure, offering incremental risk prognostication over the spectrum of heart failure progression [1,8]. Peak oxygen consumption offers strong prognostic information, but can be limited in interpretation when an adequate respiratory exchange ratio is not met. Furthermore, the relationships with intracardiac filling pressures during exercise and ventilator efficiency parameters including peak VO_2_ are not entirely known.

Incremental changes in intracardiac pressure during exercise are one of the major causes of dyspnea in patients with chronic HFrEF, and hemodynamic monitoring during exercise using invasive right heart catheterization can be used to accurately assess for these changes [4,9,10]. Although clinically informative, invasive CPET testing is both time- and resource-consuming, which may limit its widespread implementation.

The ReDS system is a novel noninvasive tool used to quantify lung fluid volume, which correlates well with intracardiac filling pressures by right heart catheterization and intraparenchymal fluid using high resolution computed tomography, when measured at rest condition [5,6,11]. The prognostic impact of ReDS values measured at rest condition was previously investigated [12].

We demonstrated only the feasibility of measuring ReDS during exercise, whereas the utility of conducting ReDS measurements during exercise as compared with other modalities remains unknown. The association between the change in ReDS during exercise and ventilator efficiency parameters also remains unknown. Of note, the patient observed with increased ReDS values during exercise had relatively preserved exercise capacity. Whether the increase in ReDS values during exercise has an independent and additional prognostic impact upon conventional ventilator efficiency parameters, including peak VO_2_, requires further time-to-event analyses. Furthermore, strategies to aggressively intervene upon the increase in ReDS values during exercise remain in need of further prospective analysis.

This is a preliminary proof-of-concept study, and has several limitations. All participants had reduced or mildly reduced ejection fraction, and the applicability of our findings to other etiologies, including heart failure with preserved ejection fraction, requires further analysis. With a small sample size and a skewed distribution of the outcome towards the null, larger scale studies are needed in order to understand the phenotypes of patients who have clinically meaningful changes in ReDS values following exercise. We did not compare the ReDS values with any other modalities that assess lung fluid amounts, and we only showed the feasibility of taking ReDS measurements during CPET. The usefulness of the ReDS system should be validated by comparison with other modalities in subsequent studies.

## 5. Conclusion

The ReDS system may be a feasible complementary tool to noninvasively assess the changes in lung fluid amounts during cardiopulmonary exercise testing. Its clinical implication needs further investigation.

## Figures and Tables

**Table 1:** Baseline characteristics.

	(*N* = 13)
Demographics	
Age, years	41 (37, 52)
Men	9 (69%)
Body mass index	23.2 (19.9, 26.9)
Etiology	
Hypertensive heart disease	2 (15%)
Ischemic heart disease	0 (0%)
Dilated cardiomyopathy	7 (54%)
Dilated phase of hypertensive cardiomyopathy	2 (15%)
Others	2 (15%)
Comorbidity	
Atrial fibrillation	3 (23%)
Diabetes mellitus	2 (15%)
History of stroke	1 (8%)
History of smoking	6 (46%)
History of heart failure admission	9 (69%)
Previous heart failure admission times	2 (1, 3)
Echocardiography data	
Left ventricular end-diastolic diameter, cm	66 (54, 77)
Left ventricular ejection fraction, %	29 (19, 40)
Left atrial diameter, mm	38 (31, 47)
Moderate or greater mitral regurgitation	1 (8%)
Moderate or greater tricuspid regurgitation	1 (8%)
Laboratory data	
Hemoglobin, g/dL	14.0 (11.9, 16.4)
Serum albumin, mg/dL	3.6 (2.9, 4.5)
Serum sodium, mEq/L	138 (136, 140)
Estimated glomerular filtration ratio, mL/min/1.73 m^2^	63.9 (47.7, 81.8)
Plasma B-type natriuretic peptide, pg/mL	139 (73, 373)
Medication	
Beta-blocker	13 (100%)
Renin-angiotensin system inhibitor	13 (100%)
Mineralocorticoid receptor antagonist	1 (8%)
Diuretics	9 (69%)
Intravenous catecholamine infusion	2 (15%)
New York Heart Association functional class (I/II/III/IV)	0/8/3/2
Cardiopulmonary exercise test parameters	
Exercise duration, min	449 (362, 506)
Work load, Watt	42 (32, 66)
Respiratory exchange ratio	1.12 (1.10, 1.14)
Peak heart rate, bpm	112 (95, 130)
Peak oxygen consumption, mL/kg/min	11.4 (10.7, 14.0)
Anaerobic threshold, mL/kg/min	8.2 (6.9, 8.7)
Minute ventilation/carbon dioxide production slope	33.1 (30.9, 36.7)
Oxygen uptake efficiency slope	1232 (828, 1410)
Peak end-tidal carbon dioxide, %	5.0 (4.8, 5.1)
Peak oxygen consumption/heart rate, mL/bpm	6.4 (5.7, 9.6)
Δ oxygen consumption/Δ work load, mL/min/Watt	7.8 (5.9, 9.6)

## Data Availability

Data are available upon reasonable requests.
